# Absent monofilament sensation in a type 2 diabetic feet

**DOI:** 10.1080/17571472.2017.1370813

**Published:** 2017-09-04

**Authors:** Julia Cheong, K. Alexiadou, Senan Devendra

**Affiliations:** a Faculty of Health Sciences, Bristol University, Bristol, UK; b Department of Diabetes, North West Thames Deanery, Watford General Hospital, Watford, UK; c Department of Acute Medicine & Endocrinology, Watford General Hospital, Watford, UK

**Keywords:** Neuropathy, diabetes, foot ulcers, amputation, falls

## Abstract

Neuropathy is a common complication which can affect up to 90% of patients with diabetes mellitus. Asymptomatic neuropathy is a common presentation. We present a case that emphasises the importance of foot screening in people with diabetes. It also highlights that patient education is key to prevent development of foot ulceration which can lead to amputations. In addition, pharmacological therapy (as per NICE guidance) can be offered for pain relief. Patients with diabetic neuropathy are at high risk of falling and sustaining fractures.

## Why this matters to me

Screening of the feet is routinely done in primary care in people with diabetes. However various actions are needed to be done when neuropathy is detected. Most patients with neuropathy may have other cardiovascular risk factors that need to be addressed. Other causes of neuropathy must be considered when investigating patients with neuropathy. Education about feet care is important in preventing amputations in the future.

## Key message

Educating patients with diabetic neuropathy about foot care is essential to prevent amputations.

## Case history

A 67 year old man with a 12 year history of Type 2 diabetes recently had a foot check with the practice nurse. His body mass index was 31 kg/m^2^. On examination, it was noted that he had absent monofilament testing in 7 out of 10 areas in both feet. No other neurological findings were noted. He is currently on metformin 1 g twice daily, gliclazide 80 mg twice daily, ramipril 10 mg once daily, and simvastatin 40 mg at night. His most recent HbA1c was 8.8% (72.7 mmol/mol). He denies any other symptoms.

## Ways of approaching the case

### Has diabetes contributed to the aetiology of neuropathy in this patient?

Microvascular complications such as diabetic neuropathy generally occur after 10 years of diagnosis with diabetes. However, diabetic neuropathy can also be observed in patients with impaired glucose tolerance or newly diagnosed Type 2 diabetes.

A full history and examination should be performed on anyone presenting with neuropathy to exclude any serious or sinister pathologies and help identify any treatable causes. A history of alcohol intake and a detailed drug history are particularly important to screen for any social risk factors associated with diabetic neuropathic pain [1].

A full neurological examination is essential to identify other pathology. The feet and legs can be tested for different sensory modalities such as vibration, fine touch, pain and proprioception. Classically, vibration and proprioception are affected to a greater degree than pinprick sensation. It should be noted that in most cases, there is little need to perform formal nerve conduction studies.

Whilst diabetes is the most likely explanation for the development of neuropathy, it is essential to identify other possible causes for the numbness and pain as these may be treatable in their own right (See Table 1) [2],[3].

**Table 1.** Summary of the different causes of peripheral neuropathy. Compiled from [2] and [3].

Other causes of peripheral neuropathy	Additional information
Alcohol	Chronic alcoholism is linked to malnutrition and deficiencies (Vitamin B12, B1)
Autoimmune	Examples include Guillain-Barré syndrome, vasculitis, sarcoidosis
Kidney failure	Other pre-renal, renal and post renal causes
Toxins	Toxins, poisons and chemicals, such as exposure to lead or mercury
Vitamin and nutrition deficiency	Vitamin B12 and/or folate deficiency, (e.g. Metformin therapy)
Chemotherapy-induced	Medication causes nerve damage, depending on dosage
Idiopathic neuropathy	Unknown causes make up to 30% of neuropathies
Hereditary disorders	Commonly: Charcot-Marie Tooth Disease and Hereditary Neuropathy with Liability to Pressure Palsies
Inflammatory and infectious causes	Lyme disease, leprosy, herpes zoster virus, hepatitis B, C, HIV/AIDS
Other	Unclassified causes such as physical injury, trauma

### What are the types of diabetic neuropathies?

Diabetic neuropathy can affect up to 90% of patients with diabetes and is regularly seen as a complication in both type 1 and type 2 diabetes [1]. There are two notable forms of diabetic polyneuropathy, based on the absence or presence of pain. In the first case, patients develop a distal symmetrical polyneuropathy. This gives rise to the classic ‘glove and stocking’ distribution of sensory loss. Distal symmetrical neuropathy affects the longest nerve fibres first and originates from the toes and feet. It gradually progresses in a proximal fashion, eventually affecting both feet and legs. This form of neuropathy is notable as there is usually an absence of symptoms. Patients may complain of numbness or tingling sensations as well as unsteadiness on their feet. A classical complaint would be as if one is walking on cotton-wool. Indeed, the ‘unsteadiness’ is a manifestation of the loss of proprioception, leading to an impairment in walking, as noted by some patients.

The other main form of diabetic polyneuropathy is painful neuropathy where patients often describe pain in the distal lower limb as sharp, burning or even shooting. The pattern of pain distribution is often symmetrical and worse at night.

An alternative clinical classification of diabetic neuropathies can be divided into two sets: symmetric polyneuropathies and asymmetric/focal and multifocal diabetic neuropathies. Table 2 summarises the details of these two categories.

**Table 2.** Clinical classification of diabetic neuropathies. From [4].

Symmetric Polyneuropathies	Asymmetric/Focal and Multifocal Diabetic Neuropathies
Where deficits are relatively fixed: • Distal sensory polyneuropathy • Autonomic neuropathy (see Table 3) • Diabetic neuropathic cachexia Symptoms may be episodic in: • Diabetic neuropathic cachexia • Hyperglycaemic neuropathy • Treatment-induced diabetic neuropathy	• Diabetic lumbosacral radiculoplexopathy (includes, diabetic amyotrophy and proximal diabetic neuropathy) • Truncal (thoracic) neuropathy • Cranial neuropathy • Limb mononeuropathy

Diabetic autonomic neuropathy can affect different organs and systems, notably the cardiovascular, gastrointestinal and urogenital functions, as illustrated in Table 3.

**Table 3.** The main repercussions of diabetic autonomic neuropathy on the cardiovascular, gastrointestinal and urogenital systems [5].

System affected	Symptoms/Manifestation
Cardiovascular	• Resting tachycardia • Exercise intolerance • Orthostatic hypotension • Silent myocardial ischaemia/cardiac denervation syndrome • Prolonged QT interval
Gastrointestinal	• Oesophageal dysmotility • Delayed gastric emptying (Gastroparesis diabeticorum) • Constipation • Diarrhoea • Faecal incontinence
Urogenital	• Neurogenic bladder (diabetic cystopathy) • Erectile dysfunction • Retrograde ejaculation • Female sexual dysfunction (e.g. vaginal lubrication loss)

### Does improving glycaemic control help prevent the progression of neuropathy?

Intensive glycaemic therapy during the DCCT study in patients with Type 1 diabetes significantly reduced the risk of diabetic peripheral neuropathy by 60% [6]. In contrast, studies in people with Type 2 diabetes only showed a moderate 5–7% improvement in neuropathy [7]. Both poor glucose control as well as rapid correction of hyperglycaemia can be associated with an increased risk of neuropathy. Acute onset of neuropathic pain can occur when a rapid improvement in glycemic control occurs. This is specified as a decrease in glycosylated HbA1c of more than 2% points over 3 months [8]. This is described as treatment-induced neuropathy in diabetes. The underlying pathophysiological mechanism is poorly understood. It has been suggested that rapid glycemic control both with and without insulin leads to haemodynamic changes (arteriovenous shunting) resulting in endoneurial hypoxia of small fibres [9].

The EURODIAB Prospective Complications Study showed that apart from glycosylated haemoglobin and the duration of diabetes, modifiable cardiovascular risk factors such as raised triglyceride levels, body mass index, smoking and hypertension can also play a role in the incidence of neuropathy [10]. Therefore, addressing these cardiovascular risk factors could potentially reduce the incidence of neuropathy.

### How should the patient be managed and what advice can he be offered?

The majority of diabetic neuropathic cases can be managed in primary care. Referral to a diabetologist or dedicated foot service would only be indicated if unusual features or active non-healing ulceration is noted upon examination. In addition, referral to a neurologist may be warranted if there are co-existing features of a systemic illness. Achieving optimum glycaemic control will help prevent any deterioration. In this case, the gliclazide dose in this patient should be gradually increased to the maximum daily dose (i.e. 160 mg twice daily). If his HbA1c still remains suboptimal, then the initiation of other oral therapies such as an SGLT-2 inhibitor (e.g. empaglifozin) may be considered.

In terms of pharmacological therapy for the symptomatic control of painful diabetic neuropathy, a choice of amitriptyline, duloxetine, gabapentin or pregabalin should be offered as initial treatment for neuropathic pain. If the initial treatment is not effective or is not tolerated, one of the remaining 3 drugs should be offered, and further switching  should be considered if the second and third drugs tried are also ineffective or not tolerated [7].

Educating the patient is of utmost importance: self-examination is often the fastest way of detecting any visible changes or injuries in the feet. The patient should be warned of the possible lack of painful sensations in the feet as a painless injury or lesion is not in itself reassuring. In this situation, patients should be prompted to seek medical advice rapidly and avoid self-treating. Appropriate footwear should be worn, such as laced shoes as they provide adequate protection if worn properly. The use of sandals and flip-flops should be discouraged, as well as walking barefoot since these can expose the feet  to sharp foreign objects and hence increase the risk of injury. Patients should also be discouraged from warming their feet near a fire or with a hot water bottle as this can cause unnoticed burns.

Several studies have demonstrated an increased risk of falls and fall -associated fractures in people with diabetes and with  foot deformities, reduced sensation or ulcers [11,12] Increased attention towards falls and falls assessment is therefore crucial and should  constitute an area of focus for care providers.

As discussed, diabetic neuropathy is the most important risk factor for ulceration and progression to amputation, mainly due to being unaware of injury to the foot. Ulceration or infection in a diabetic foot should be taken seriously and referral to a dedicated foot service is usually warranted to minimise the risk of amputation.

## Further reading

Edmonds ME, Foster AVM, and Sanders LJ. A practical manual of diabetic foot care. 2004. Blackwell Publishing.

## Patient information leaflet

https://www.diabetes.org.uk/putting-feet-first

## Governance

This work is based on a case discussed at the Ealing Integrated Care Programme.

## Disclosure statement

All authors declare that the answer to the questions on your competing interest form are all [No] and therefore have nothing to declare.
